# On Computational Aspects of Krawtchouk Polynomials for High Orders

**DOI:** 10.3390/jimaging6080081

**Published:** 2020-08-13

**Authors:** Basheera M. Mahmmod, Alaa M. Abdul-Hadi, Sadiq H. Abdulhussain, Aseel Hussien

**Affiliations:** 1 Department of Computer Engineering, University of Baghdad, Baghdad 10071, Iraq; basheera.m@coeng.uobaghdad.edu.iq (B.M.M.); alaa.m.abdulhadi@coeng.uobaghdad.edu.iq (A.M.A.-H.); 2 Department of Computer Science, Liverpool John Moores University, Liverpool L3 3AF, UK; a.hussien@ljmu.ac.uk

**Keywords:** Krawtchouk polynomials, Krawtchouk moments, high order polynomials, propagation error, image reconstruction analysis

## Abstract

Discrete Krawtchouk polynomials are widely utilized in different fields for their remarkable characteristics, specifically, the localization property. Discrete orthogonal moments are utilized as a feature descriptor for images and video frames in computer vision applications. In this paper, we present a new method for computing discrete Krawtchouk polynomial coefficients swiftly and efficiently. The presented method proposes a new initial value that does not tend to be zero as the polynomial size increases. In addition, a combination of the existing recurrence relations is presented which are in the *n*- and *x*-directions. The utilized recurrence relations are developed to reduce the computational cost. The proposed method computes approximately 12.5% of the polynomial coefficients, and then symmetry relations are employed to compute the rest of the polynomial coefficients. The proposed method is evaluated against existing methods in terms of computational cost and maximum size can be generated. In addition, a reconstruction error analysis for image is performed using the proposed method for large signal sizes. The evaluation shows that the proposed method outperforms other existing methods.

## 1. Introduction

Discrete orthogonal moments, simply moments, are utilized as a feature descriptor in several fields such as signal processing and computer vision [1]. Moments, mathematically, are formed from the projection of a signal on the discrete orthogonal polynomial basis to ensure non-redundancy of the feature set [2,3]. Tchebichef, Hahn, Charlier, and Krawtchouk polynomials are types of polynomials that are used to generate discrete moments. Amongst them, Krawtchouk can express an image locally [2]. Beside, discrete Krawtchouk polynomials (DKPs) have been combined with other polynomials to enhance the performance of the resulted polynomial by adding the localization property of the DKP such as discrete Krawtchouk–Tchebichef polynomials [4], Squared Tchebichef–Krawtchouk polynomials [5], and Squared Krawtchouk–Tchebichef polynomials [6]. Krawtchouk polynomials and its hybrid forms have been utilized in different applications such as speech enhancement [7], shot boundary detection [8,9], and information hiding [10]. The DKP has a parameter (*p*) that controls the shifting direction of the features [11]. The parameter *p* at 0.5 has a special case because it equally divides the moment plane into four portions and makes the feature extraction more simple than other values of parameter *p*.

Many efforts have been conducted to compute the DKP coefficients (DKPCs) using three-term recurrence algorithm (TTRA). The TTRA is employed as a replacement to the hypergeometric series and gamma functions because these functions are time-consuming. Yap et al. [11] presented TTRA in the *n*-direction to compute DKPCs. Koekkoek et al. [12] presented the TTRA in the *x*-direction to generate DKPCs. However, when the image size becomes large, DKPCs shows instability because of the numerical propagation errors. The method presented in [13] shows that a reduction in the computation of recurrence times is the key to reduce the numerical error. However, the existing methods suffer from the problem of initial value which tends to be zero as the polynomial-size increases [13]. To overcome this problem, this paper proposes a new method to compute the DKPCs efficiently and swiftly. The proposed method investigated a new location to compute the initial value which is then used to compute the rest of the DKPCs. Based on the location of the initial value, a new TTRA is presented to compute the DKPCs with a reduction in the computation of polynomials coefficients.

## 2. Preliminaries

In this section, the definitions DKPs are presented as well as the recurrence relation which are employed in the proposed method. The *n*-th order of the weighted and normalized DKP is defined as follows [12]:

(1) 𝓚npx=N−1xN−1np1−pn+x×2F1−n,−x−N+1|1p,n=0,1,2,…,N−1,andx=0,1,2,…,N−1,N>0;p∈(0,1),

where ${}_{2}F_{1}$ denotes the hypergeometric series and is defined as:

(2) $${}_{2}F_{1}\left(\begin{array}{c} -n,-x\\ -N+1 \end{array}|\;\frac{1}{p}\right)=\sum_{k=0}^{\infty }\frac{{\left(-n\right)}_{k}{\left(-x\right)}_{k}}{{\left(-N+1\right)}_{k}\;k!}{\left(\frac{1}{p}\right)}^{k},$$

where ${\left(\cdot \right)}_{k}$ represents the rising factorial defined as:

(3) $${\left(a\right)}_{k}=\frac{\Gamma (a+k)}{\Gamma (a)}.$$

**Figure 1:**
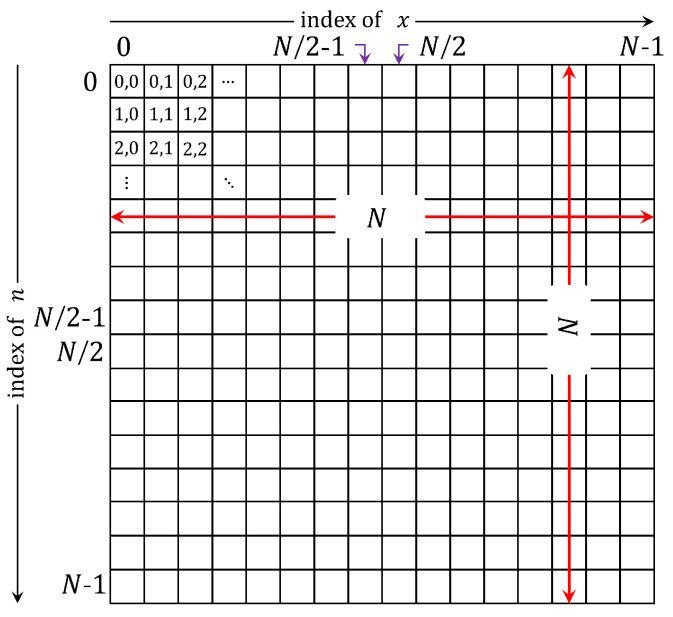
The 2D array parameters of DKPCs.

DKPs are generally a two-dimensional array with three parameters as shown in Figure 1, which are: (1) the size of the array N×N, (2) the polynomial order parameter (*n*), and (3) the polynomial index parameter (*x*).

The computation of the DKPCs using Equation (1) is considered computationally cost due to the usage of hypergeometric and gamma functions. Therefore, the three-term recurrence algorithm (TTRA) is employed for computing the DKPCs [14]. Two types of TTRAs are introduced: TTRAs in the *x*- and *n*-directions.

The TTRA in the n-direction is given by [11]:

(4) $$\begin{array}{cc} p\left(n-N\right){𝓚}_{n+1}^{p}\left(x\right) & =A\left(Np-2np+n-x\right){𝓚}_{n}^{p}\left(x\right)-B\left(1-p\right){𝓚}_{n-1}^{p}\left(x\right),\\ n= & 1,2,\ldots ,N-2,\;\mathrm{and}\;\\ x= & 0,1,\ldots ,N-1, \end{array}$$

where

(5) $$\begin{array}{c} \begin{array}{cc} A & =\sqrt{\frac{\left(1-p)(n+1\right)}{p(N-n)}}\\ B & =\frac{\left(1-p\right)}{p}\sqrt{\frac{(n+1)n}{\left(N-n)(N-n+1\right)}}\;\;. \end{array} \end{array}$$

In this algorithm, the polynomial coefficients are computed by employing the coefficients at the orders n−1 and n−2.

The TTRA in the *x*-direction is given by [12]:

(6) $$\begin{array}{cc} C{𝓚}_{n}^{p}\left(x+1\right) & =D{𝓚}_{n}^{p}\left(x\right)+E{𝓚}_{n}^{p}\left(x-1\right),\\ n= & 1,2,\ldots ,N-1,\;\mathrm{and}\;\\ x= & 1,2,\ldots ,N/2-2, \end{array}$$

(7) $$\begin{array}{c} \begin{array}{cc} C & =\sqrt{p(N-x-1)(1-p)(x+1)},\\ D & =-n+p(N-x-1)+x(1-p),\\ E & =\sqrt{x(1-p)p(N-x)}\;\;. \end{array} \end{array}$$

In this algorithm, the DKPCs are computed by considering the values of the coefficients at the indices x−1 and x−2.

## 3. Proposed Recurrence Algorithm

**Figure 2:**
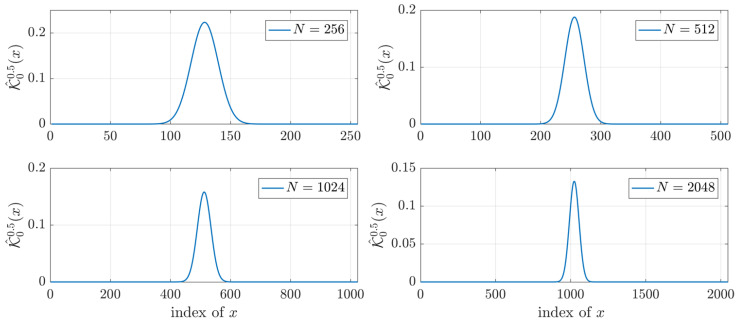
Plot of ${𝓚}_{0}^{0.5}\left(x\right)$ for different values of polynomial size.

Computing the initial value is considered important and impacts the values of DKPCs. Previous algorithms failed to compute DKP for high order because the location where initial value computed goes to zero which in turn makes the rest of the values polynomial zero out. Thus, unlike the existing algorithm which considers ${𝓚}_{0}^{p}\left(0\right)$ as the initial values. Figure 2 shows the results of the ${𝓚}_{0}^{0.5}\left(x\right)$ for different values of polynomial size. From Figure 2, it is clear that when x=0, the values of ${𝓚}_{0}^{0.5}\left(x\right)$ becomes very small and in several environments considered zero. On the other hand, the value of ${𝓚}_{0}^{0.5}\left(x\right)$ at x=N/2−1 always large values.

The proposed initial value is computed at n=0 and x=N/2−1. From Equation (1), the initial value can be written as follows:

(8) $${𝓚}_{0}^{0.5}\left(\frac{N}{2}-1\right)=\sqrt{\frac{4{\left(\frac{1}{4}\right)}^{N/2}\Gamma \left(N\right)}{N\;\Gamma {\left(N/2\right)}^{2}}}.$$

**Figure 3:**
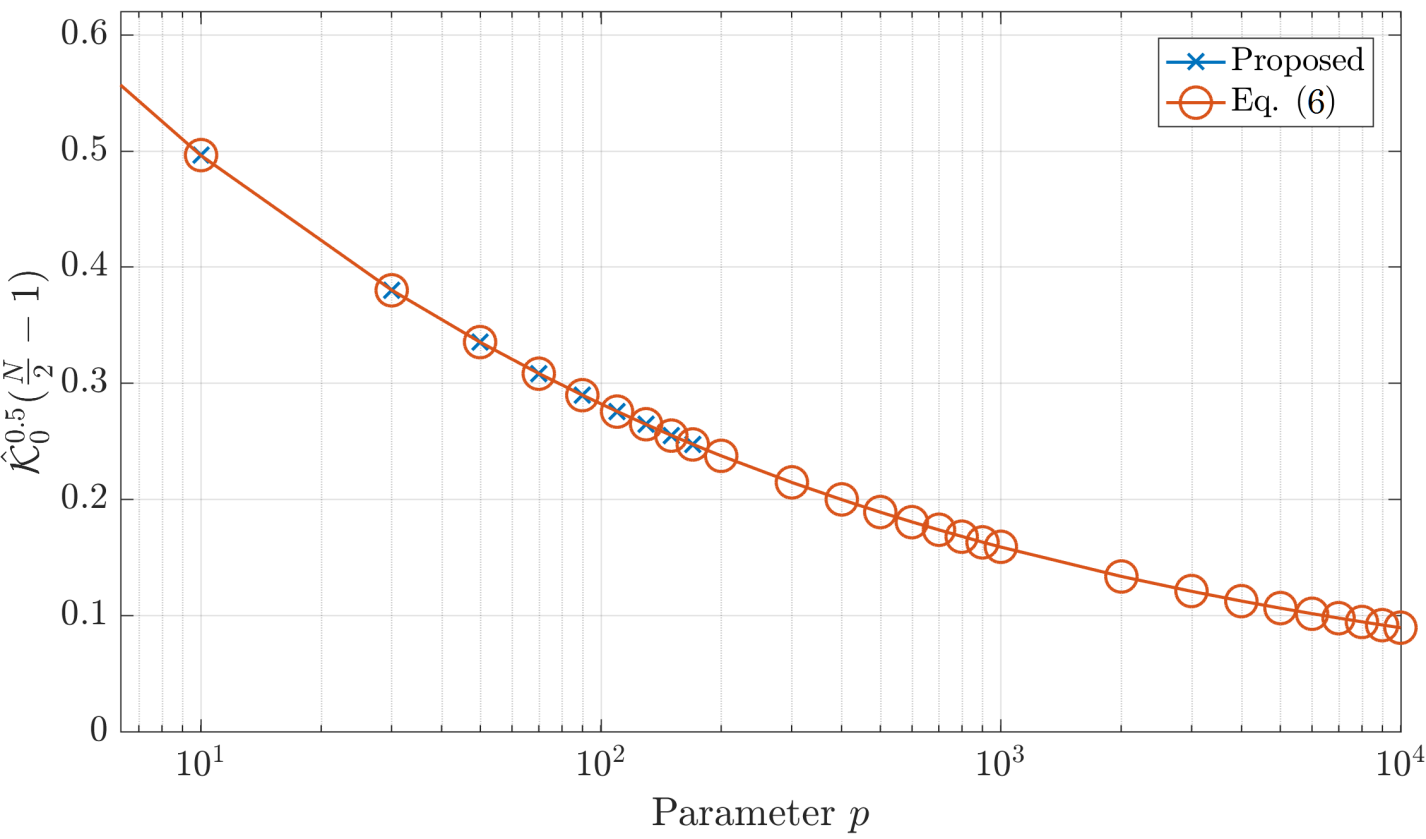
The plot of ${𝓚}_{0}^{0.5}\left(\frac{N}{2}-1\right)$ using the proposed formula (Equations (9)) and (8).

The problem with Equation (8) is that the gamma function, Γ(·), produces very large numbers and produces infinity in several environments such as MATALB and python. To overcome this problem, natural logarithmic gamma function, lnΓ(·), can be utilized. Thus, Equation (8) can be written as follows:

(9) $${𝓚}_{0}^{0.5}\left(\frac{N}{2}-1\right)=\sqrt{exp\left(ln(4)+ln\Gamma (N)-2ln\Gamma (N/2)-ln(N)-0.5N\;ln(4)\right)},$$

where ln(·) represents the natural logarithmic function lnΓ(·) represents the logarithmic gamma function. The maximum limit of each function is presented in Figure 3 which clearly shows that the proposed formula can compute the initial values for a wide range of polynomial size.

**Figure 4:**
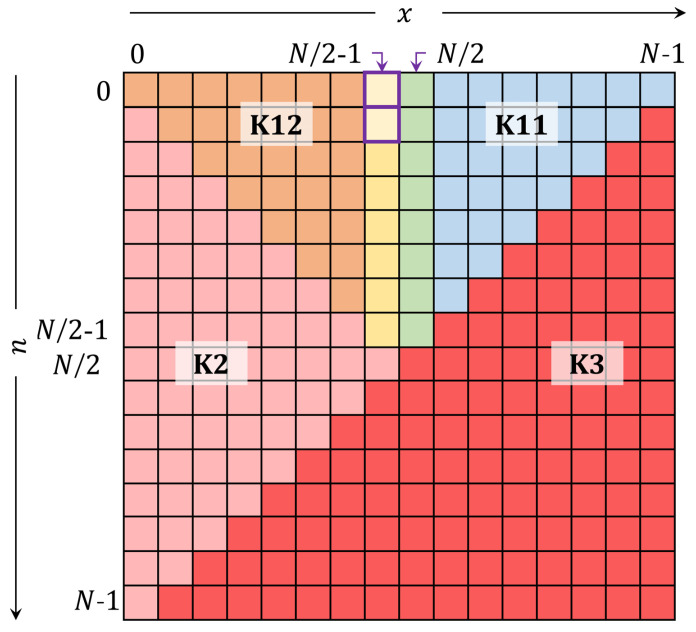
The partitions of the DKP plane.

The plane of the DKP is partitioned as shown in Figure 4. After finding a computable initial value, the value of the coefficient at n=1 and x=N/2−1 need to be computed. The coefficient of ${𝓚}_{1}^{0.5}\left(\frac{N}{2}-1\right)$ is computed as follows:

(10) $${𝓚}_{1}^{0.5}\left(\frac{N}{2}-1\right)=-\frac{1}{\sqrt{N-1}}{𝓚}_{0}^{0.5}\left(\frac{N}{2}-1\right).$$

It is noteworthy mentioning that the plane of the DKP is partitioned as shown in Figure 4. After computing the four coefficients, the TTRA in the *n*-direction can be employed to compute the values in the range n=0,1,…,N/2−1 and x=N/2−1,N/2. However, a reduced form of the TTRA in the *n*-direction is presented and used. The reduced form of the TTRA is defined as:

(11) $$\begin{array}{cc} {𝓚}_{n+1}^{0.5}\left(x\right) & =\alpha_{1}{𝓚}_{n}^{0.5}\left(x\right)+\alpha_{2}{𝓚}_{n-1}^{0.5}\left(x\right),\\ \alpha_{1} & =\frac{N-1-2x}{\sqrt{\left(N-1-n)(n+1\right)}},\\ \alpha_{2} & =\sqrt{\frac{n(N-n)}{\left(N-n-1)(n+1\right)}},\\ n & =1,2,\ldots ,N/2-1,\;\mathrm{and}\;\\ x & =N/2-1. \end{array}$$

To compute the values in the range n=0,1,…,N/2−1 and x=N/2, the following symmetry relation is utilized [13]:

(12) $$\begin{array}{c} {𝓚}_{n}^{0.5}\left(\frac{N}{2}\right)={\left(-1\right)}^{n}{𝓚}_{n}^{0.5}\left(\frac{N}{2}-1\right),\\ n=1,2,\ldots ,N/2-1. \end{array}$$

Next, the coefficients of the DKP are computed in the range n=0,1,…,N/2−1 and x=N/2,…,N−n−2 (region K11 in Figure 4) using the proposed reduced form in the *x*-direction TTRA as follows:

(13) $$\begin{array}{cc} {𝓚}_{n}^{0.5}\left(x+1\right) & =\frac{\beta_{1}}{\beta_{x+1}}{𝓚}_{n}^{0.5}\left(x\right)-\frac{\beta_{x}}{\beta_{x+1}}{𝓚}_{n}^{0.5}\left(x-1\right),\\ \beta_{1} & =(N-1-2n),\\ \beta_{x} & =\sqrt{\left(N-(x+1)\right)\left(x+1\right)}\;\;. \end{array}$$

The coefficient in the range n=0,1,…,N/2−1 and x=n,…,N/2−1 (region K12 in Figure 4) is computed using symmetry relation [13] as follows:

(14) $${𝓚}_{n}^{0.5}\left(x\right)={\left(-1\right)}^{n}{𝓚}_{n}^{0.5}\left(N-x-1\right).$$

The coefficients in the range x=0,1,…,N/2−1 and n=x+1,x+2,…,N−x−1 (region K2 in Figure 4) are computed using the following symmetry relation [13]:

(15) $${𝓚}_{n}^{0.5}\left(x\right)={𝓚}_{x}^{0.5}\left(n\right).$$

The coefficients in the range x=0,1,…,N−1 and n=N−x−1,…,N−1 (region K3 in Figure 4) are computed using the following symmetry relation [13]:

(16) $${𝓚}_{n}^{0.5}\left(x\right)={\left(-1\right)}^{N-n-x-1}{𝓚}_{N-n-1}^{0.5}\left(N-x-1\right).$$

**Figure 5:**
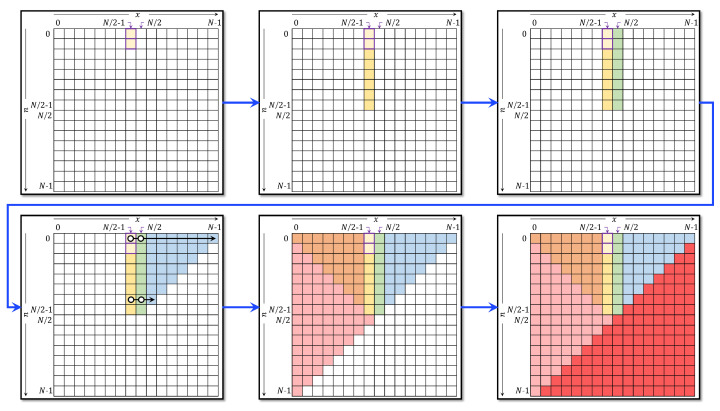
The steps used to compute DKPCs using the proposed recurrence algorithm.

The steps of the method are depicted in Figure 5 and can be summarized as follows:

1. The Initial value ${𝓚}_{0}^{0.5}\left(N/2-1\right)$ is computed using Equation (9).
2. The value of ${𝓚}_{0}^{0.5}\left(N/2\right)$ is computed from ${𝓚}_{0}^{0.5}\left(N/2-1\right)$ using Equation (10).
3. The first set of initial used for TTRA is computed in the range of n=2,3,…,N/2−1 and x=N/2−1 using Equation (11).
4. The first set of initial used for TTRA is computed in the range of n=2,3,…,N/2−1 and x=N/2 using Equation (12).
5. The modified recurrence algorithm, Equation (13), is used to compute the values of the coefficients in the range n=0,1,…,N/2−1 and x=N/2,…,N−n−2. It should be noted that, for each *x*, when ${𝓚}_{n}^{0.5}\left(x+1\right)<10^{-7}$ and ${𝓚}_{n}^{0.5}\left(x\right)<10^{-5}$, the recurrence relation is terminated and *n* is increased by 1.
6. The rest of the DKPCs are computed using symmetry relations in Equations (14)–(16).

## 4. Performance Evaluation of the Proposed Method

**Figure 6:**
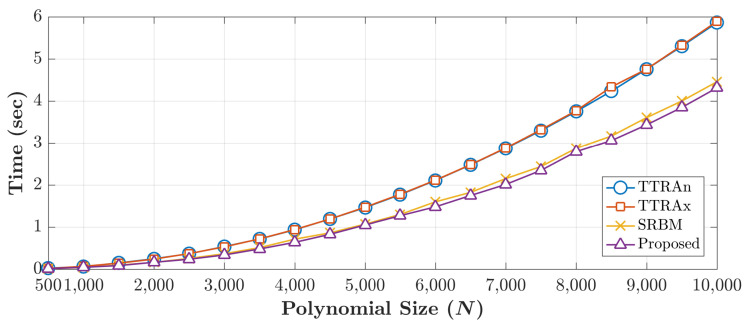
Computation time of the proposed and existing methods.

A comparison with the existing methods is performed based on two criteria: computation cost and maximum generated size. A comparison of computation time is performed for different polynomials size. The existing algorithms are the TTRA in the *n*-direction (TTRAn) [11], TTRA in the *x*-direction (TTRAx) [12], and symmetry relation-based method (SRBM) [13]. The execution time is carried out using Krawtchouk parameter p=0.5 and different DKP sizes. Figure 6 shows the execution time required for each algorithm to generate DKPCs with a size of N×N. For each method, average execution time for 10 runs is reported in Figure 6.

From Figure 6, it can be noticed that the execution times TTRAn [11] and TTRAx [12] are approximately identical because both of the algorithms are computing 50% of coefficients using the recurrence algorithm. TTRAn employs Equation (4) and TTRAx employs Equation (6). The rest of the coefficients are computed using similarity relation. For SRBM [13], only 12.5% of the coefficients are computed; thus, it shows less execution time when compared to TTRAn and TTRAx.

Obviously, in the proposed method, the average execution time to generate DKPCs is less than existing methods. This achievement is due to: (1) the proposed method computed only 12.5% of the DKPCs, and (2) a reduced complexity forms of the existing recurrence relations are introduced (Equations (11) and (13)). To affirm the results of the proposed algorithm, the improvement ratio of the execution time is measured. The improvement of the proposed algorithm to SRBM is ∼3.7%; while the improvement ratio over the TTRAn and TTRAx is ∼30%. Accordingly, it can be inferred that the proposed algorithm outperforms the existing methods in terms of the execution time.

The proposed method is also evaluated in terms of maximum size can be generated and compared to that of the existing methods. For each method, the maximum size is found using the procedure presented in [13]:

1. A test image, I, is used.
2. The test image is resized to a small size $N_{s}\times N_{s}$,
3. DKP, R, is generated with signal size and order of ($N_{s}\times N_{s}$),
4. The moment, M, is computed using $M=R\times I\times R^{T}$,
5. The test image is then reconstructed back from the moment domain using $I_{r}=R^{T}\times M\times R$,
6. The normalized mean square error (NMSE) is computed between I and $I_{r}$,
7. If the $NMSE<2.5\times 10^{-3}$, the size of the image and DKP is increased by 2, i.e., $N_{s}=N_{s}+2$ and repeat step 1 to 7.
8. If the $NMSE>2.5\times 10^{-3}$, the maximum size can be generated by the polynomial is reached and reported.
9. The maximum size considered in the experiment is set to 12,288.

where the NMSE is computed as follows:

(17) $$NMSE=\frac{\sum_{x,y}{\left(I\left(x,y\right)-I_{r}\left(x,y\right)\right)}^{2}}{\sum_{x,y}{\left(I(x,y)\right)}^{2}}.$$

**Figure 7:**
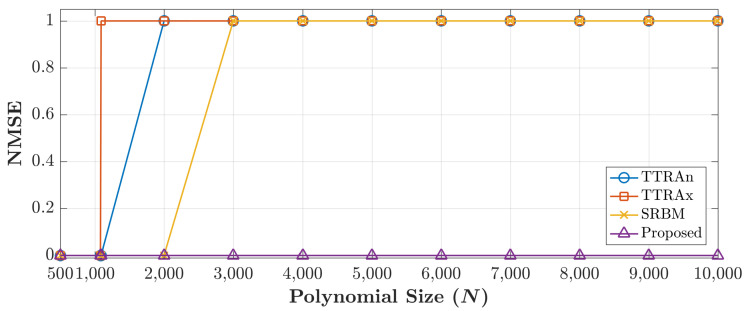
Reconstruction error of the proposed and existing methods.

Note that, the cameraman image is considered in the experiment as the test image. From the previous experiment, the maximum size of the TTRAn is 1090, TTRAx is 1080, SRBM is 2140, and for the proposed method is 12,288 (12K). The experiment reveals that the proposed method outperforms the existing method and the improvement factor is ∼5.7 times greater than SRBM and ∼11.3 times greater than TTRAn and TTRAx. To confirm the performance of the proposed algorithm, the reconstruction error in terms of the NMSE for all cases presented in Figure 6 are shown in Figure 7.

**Figure 8:**
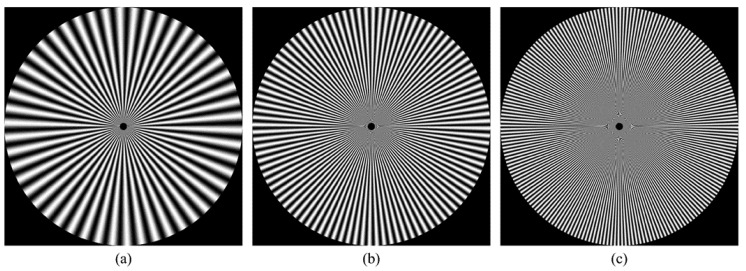
Sinusoidal Siemens star: (**a**) ω=50, (**b**) ω=100, (**c**) ω=200.

For more clarification regarding the ability of the proposed method, a reconstruction error analysis is performed for the proposed method to test its accuracy. The reconstruction error analysis is performed using a reconstruction error Sinusoidal Siemens star which is utilized to examine the resolution of optical systems and printers. Sinusoidal Siemens star involves of sinusoidal oscillations patterns in a polar coordinate system such that the spatial frequency varies for concentric circles of different sizes. The Sinusoidal Siemens star is defined as [15]:

(18) I(θ)=a+bsin(ωθ−ϕ).

where *I* represent the intensity represented by a sinusoidal function with an angle θ. In addition, a, b, ω, and ϕ are the intensity mean, the intensity amplitude, the number of cycles, and the phase offset, respectively. Figure 8 shows Sinusoidal Siemens star with different values of number of cycles ω which are ω=50,100,and200. In the experiment, the parameters of the sinusoidal Siemens star are considered as follows a=0, b=255, ϕ=0, and ω=200.

**Figure 9:**
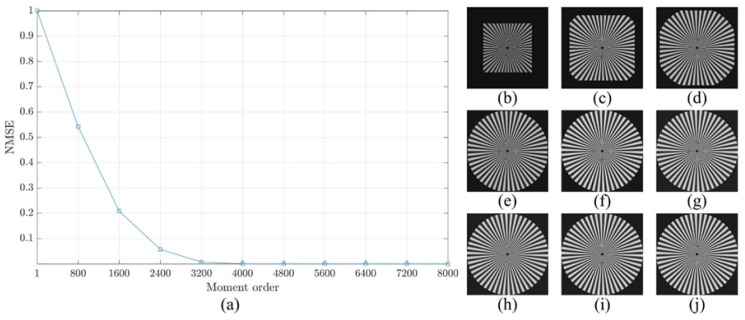
Construction analysis for the proposed method using sinusoidal Siemens start with ω=50. (**a**) NMSE, image reconstructed using number of moment equal to (**b**) 800, (**c**) 1600, (**d**) 2400, (**e**) 3200, (**f**) 4000, (**g**) 4800, (**h**) 5600, (**i**) 6400, and (**j**) 7200.

**Figure 10:**
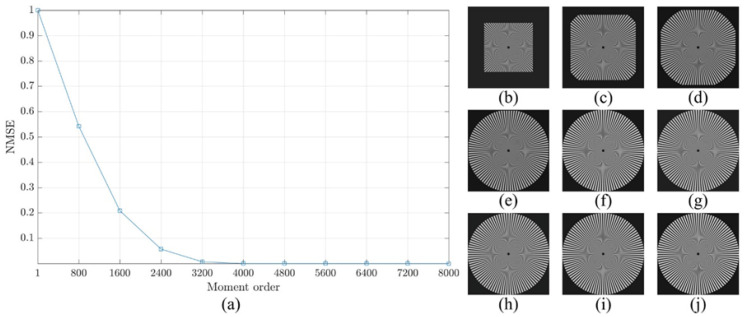
Construction analysis for the proposed method using sinusoidal Siemens start with ω=100. (**a**) NMSE, image reconstructed using number of moment equal to (**b**) 800, (**c**) 1600, (**d**) 2400, (**e**) 3200, (**f**) 4000, (**g**) 4800, (**h**) 5600, (**i**) 6400, and (**j**) 7200.

**Figure 11:**
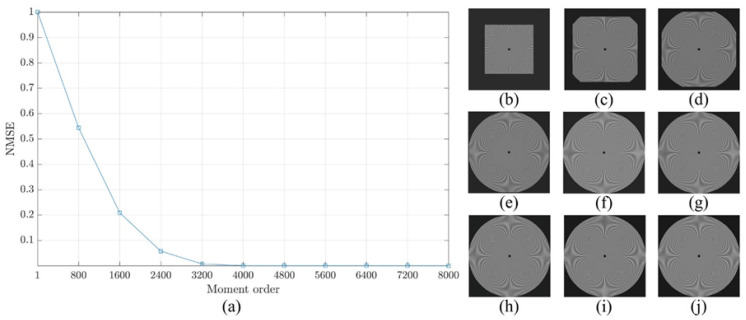
Construction analysis for the proposed method using sinusoidal Siemens start with ω=200. (**a**) NMSE, image reconstructed using number of moment equal to (**b**) 800, (**c**) 1600, (**d**) 2400, (**e**) 3200, (**f**) 4000, (**g**) 4800, (**h**) 5600, (**i**) 6400, and (**j**) 7200.

The reconstruction analysis using the proposed method is performed for an image with a size of 8000×8000. First, the image is transformed into the moment domain of the DKP. Then, the image is reconstructed using a limited number of moments. The number of moments is increased by a step of 800 until the image is fully reconstructed using the total number of moments. The obtained results are shown in Figure 9, Figure 10 and Figure 11. From the figures, it is clear that the proposed method is able to generate a stable DKP with high order. For instance, the image reconstruction analysis shows that the NMSE closes to zero when the moment order reaches 4000×4000, i.e., the DKP can represent the signal using only 50% of the moments; moreover, the spokes of sinusoidal Siemens star are never touched or overlap. This led to the proposed method to compute DKP has remarkable performance and satisfy the orthogonality condition without distortion.

**Figure 12:**
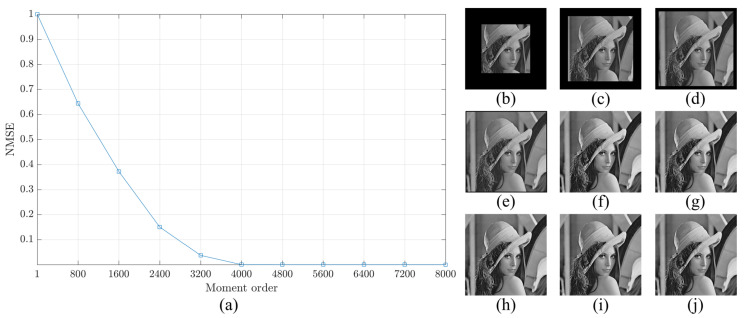
Construction analysis for the proposed method using Lena image. (**a**) NMSE, image reconstructed using number of moment equal to (**b**) 800, (**c**) 1600, (**d**) 2400, (**e**) 3200, (**f**) 4000, (**g**) 4800, (**h**) 5600, (**i**) 6400, and (**j**) 7200.

In addition to Sinusoidal Siemens star, the well known image of Lena is utilized for reconstruction analysis. The Lena image is resized to obtain Lena image with a size of 8000×8000. The same procedure for Sinusoidal Siemens is performed for Lena image. The result is shown in Figure 12. The result clearly confirm that the proposed method is able to generate a stable DKP with high order.

To sum up, the proposed method is able to compute DKP accurately and satisfy the orthogonality condition without distortion for large signal size and order.

## 5. Conclusions

This paper presents a new method for efficiently computing the DKPCs. The presented method is based on the combination of the existing recurrence methods as well as a new initial value formula with values not tend to zero. The initial values and the combined recurrence methods are utilized to compute the coefficients of the DKP in a specified portion. Then, the recurrence relations are employed to compute the rest coefficients of the DKP for other portions. The results show that the proposed method has significantly less computational cost when compared to the existing methods. In addition, the proposed method has the ability to minimize the propagation errors which in turn makes it able to generate DKP with high order and size of 12,288.

## Abbreviations

The following abbreviations are used in this manuscript:

DKP	discrete Krawtchouk polynomials
DKPC	discrete Krawtchouk polynomial Coefficient
TTRA	three-term recurrence algorithm
TTRAn	three-term recurrence algorithm in the *n*-direction
TTRAx	three-term recurrence algorithm in the *x*-direction
SRBM	symmetry relation-based method
NMSE	normalized mean square error
